# Mitochondrial and Nuclear Genes Suggest that Stony Corals Are Monophyletic but Most Families of Stony Corals Are Not (Order Scleractinia, Class Anthozoa, Phylum Cnidaria)

**DOI:** 10.1371/journal.pone.0003222

**Published:** 2008-09-16

**Authors:** Hironobu Fukami, Chaolun Allen Chen, Ann F. Budd, Allen Collins, Carden Wallace, Yao-Yang Chuang, Chienhsun Chen, Chang-Feng Dai, Kenji Iwao, Charles Sheppard, Nancy Knowlton

**Affiliations:** 1 Seto Marine Biological Laboratory, Field Science Education and Research Center, Kyoto University, Shirahama, Wakayama, Japan; 2 Biodiversity Research Centre, Academia Sinica, Nangang, Taipei, Taiwan; 3 Institute of Oceanography, National Taiwan University, Taipei, Taiwan; 4 Department of Geoscience, University of Iowa, Iowa City, Iowa, United States of America; 5 National Systematics Laboratory, NOAA Fisheries Service, National Museum of Natural History, MRC 153, Smithsonian Institution, Washington, D. C., United States of America; 6 Museum of Tropical Queensland, Townsville, Australia; 7 Akajima Marine Science Laboratory, Zamami-son, Okinawa, Japan; 8 Department of Biological Sciences, University of Warwick, Coventry, United Kingdom; 9 Center for Marine Biodiversity and Conservation, Scripps Institution of Oceanography, University of California San Diego, La Jolla, California, United States of America; 10 Department of Invertebrate Zoology, National Museum of Natural History, Smithsonian Institution, MRC 163, Washington, D. C., United States of America; Centre for DNA Fingerprinting and Diagnostics, India

## Abstract

Modern hard corals (Class Hexacorallia; Order Scleractinia) are widely studied because of their fundamental role in reef building and their superb fossil record extending back to the Triassic. Nevertheless, interpretations of their evolutionary relationships have been in flux for over a decade. Recent analyses undermine the legitimacy of traditional suborders, families and genera, and suggest that a non-skeletal sister clade (Order Corallimorpharia) might be imbedded within the stony corals. However, these studies either sampled a relatively limited array of taxa or assembled trees from heterogeneous data sets. Here we provide a more comprehensive analysis of Scleractinia (127 species, 75 genera, 17 families) and various outgroups, based on two mitochondrial genes (cytochrome oxidase I, cytochrome b), with analyses of nuclear genes (ß-tubulin, ribosomal DNA) of a subset of taxa to test unexpected relationships. Eleven of 16 families were found to be polyphyletic. Strikingly, over one third of all families as conventionally defined contain representatives from the highly divergent “robust” and “complex” clades. However, the recent suggestion that corallimorpharians are true corals that have lost their skeletons was not upheld. Relationships were supported not only by mitochondrial and nuclear genes, but also often by morphological characters which had been ignored or never noted previously. The concordance of molecular characters and more carefully examined morphological characters suggests a future of greater taxonomic stability, as well as the potential to trace the evolutionary history of this ecologically important group using fossils.

## Introduction

Molecular analyses have been used to study higher-level relationships among the Scleractinia only comparatively recently, in part because of technical difficulties. Rates of molecular evolution in mitochondrial genes are extremely slow in anthozoans [1], and finding informative nuclear markers is generally challenging. Even so, results to date have revolutionized our understanding of relationships, suggesting that first, traditional subordinal classifications are largely unsupported by molecular data [2]–[4]; second, many families are not monophyletic [2]–[7]; and third, the order Corallimorpharia, an anthozoan group lacking skeletons, may be imbedded in the skeleton-possessing scleractinians [8], but see [9] and [10]. Despite these findings, it remains the case that no study to date has examined the phylogeny of Scleractinia using a consistent combination of genes for most genera (including both Pacific and Atlantic representatives) across most families. Here we provide such an analysis, focusing on those scleractinian taxa with large numbers of zooxanthellate species, as well as members of the Corallimorpharia and other anthozoan outgroups.

**Figure 1:**
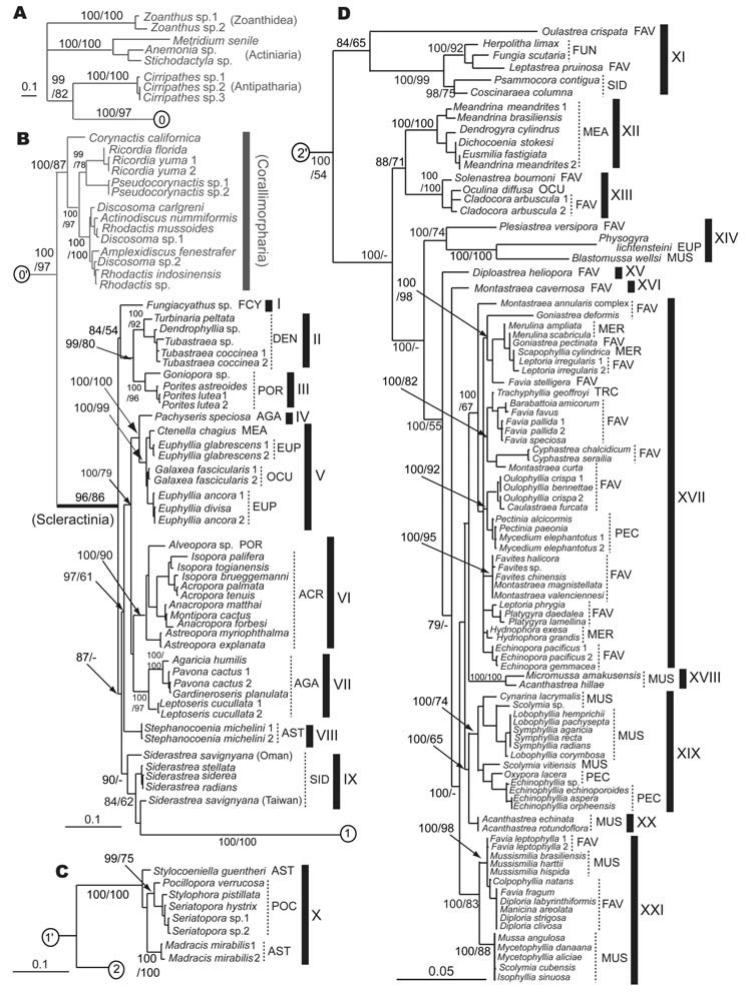
Phylogenetic relationships among scleractinian (mostly zooxanthellate) corals and outgroups. Topology was inferred by Bayesian analysis, based on combined mitochondrial *cox1* and *cob* DNA sequences. Numbers on main branches show percentages of Bayesian probability (>70%) and bootstrap values (>50%) in ML analysis. Dashes mean bootstrap values <50% in ML. Numbers in circles show the connection of trees from A to D; for example, 1′ in circle continues directly from 1 in circle. Bars in black indicate possible new family level groupings (see also Supporting Online Information Table 2). Numbers (1, 2) following species names indicate that different colonies of the species had different haplotypes. A. outgroups, B. complex corals and corallimorpharians, C. the family Pocilloporidae, D. robust corals. ACR: Acroporidae, AGA: Agariciidae, AST: Astrocoeniidae, DEN: Dendrophylliidae, EUP: Euphylliidae, FAV: Faviidae, FCY: Fungiacyathidae, FUN: Fungiidae, MEA: Meandrinidae, MER: Merulinidae, MUS: Mussidae, PEC: Pectiniidae, POC: Pocilloporidae, POR: Poritidae, OCU: Oculinidae, SID: Siderastreidae, TRC: Trachyphylliidae.

**Figure 2 pone-0003222-g002:**
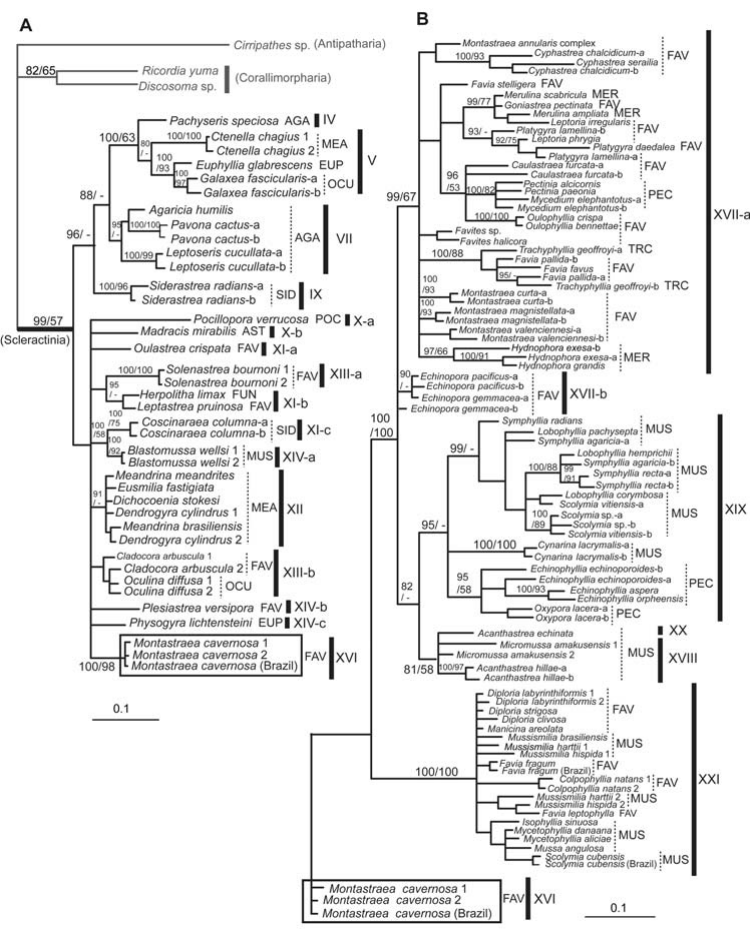
Bayesian tree based on the tubulin gene for a subset of corals shown in Fig. 1. Letter (a, b) after species names indicates that different alleles were obtained from a single coral sample; see Fig. 1 legend for other labeling conventions. A. Phylogenetic relationships among complex corals and some robust corals. B. Phylogenetic relationships within robust corals. Note that the same data for the *Montastraea cavernosa* clade (in box) were used in both trees.

**Figure 3:**
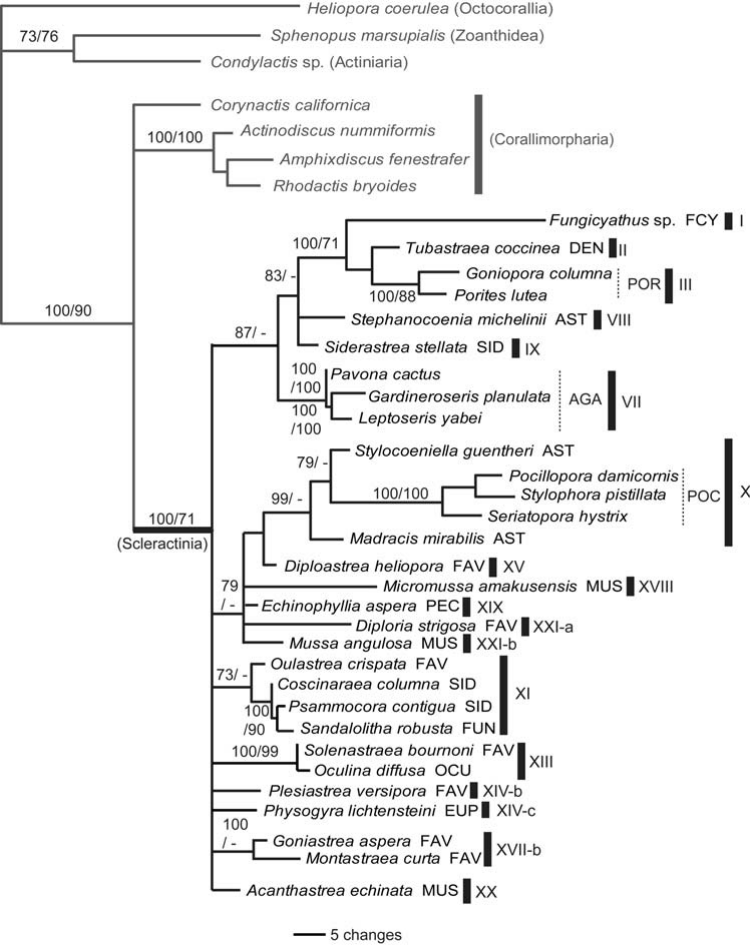
Bayesian tree based on the rDNA gene for a subset of the scleractinian corals analyzed in Fig. 1. Labeling conventions as in previous figures.

We analyzed 127 species in the order Scleractinia (Online Supporting Information, Table S1), representing 75 genera and 17 families, 16 of which have important reef-building species. This represents a substantial expansion of a previous study [6] that considered only seven families of robust corals. We included a member of the azooxanthellate family Fungiacyathidae because of its possibly close relationship with corallimorpharians [11], but did not analyze members of the largely azooxanthellate Rhizangiidae and Caryophylliidae or other exclusively azooxanthellate families studied by others [12], [13], as our focus was on reef-building groups. To test the recently suggested hypothesis that the skeletonless Corallimorpharia are imbedded within the Scleractinia, we analyzed seven genera in this order, including members of both the Corallimorphidae and Discosomatidae [14]. Outgroups consisted of members of the hexacorallian orders Antipatharia, Actiniaria, and Zoanthidea, which are the closest relatives of the Scleractinia and Corallimorpharia [11]. The initial analyses were based on two mitochondrial genes: cytochrome oxidase I (*cox1*) and cytochrome b (*cob*). A subset of taxa was analyzed using two nuclear gene regions: ß-tubulin and/or parts of the nuclear ribosomal genes (it was not possible to amplify both nuclear genes for all taxa). The nuclear analyses were targeted to test mitochondrial results that strongly contradicted traditional morphological classification. Unless noted otherwise, all conclusions are supported by both the mitochondrial dataset (Fig. 1) and at least one of the two nuclear datasets (Figs. 2, 3).

## Results

### Phylogenetic relationships within the Scleractinia

Our molecular results are shown in Figures 1–3, and summarized below (see also Table S2 in Online Supporting Information). Overall, of the 16 traditional families of scleractinian corals with reef-building genera that we analyzed, at least 11 are polyphyletic as currently defined: Mussidae, Faviidae, Pectiniidae, Merulinidae, Siderastreidae, Astrocoeniidae, Euphylliidae, Meandrinidae, Poritidae, Agariciidae, and Oculinidae. Five of these (Oculinidae, Euphylliidae, Meandrinidae, Siderastreidae, Astrocoeniidae) have members placed in both of the highly divergent “complex” and “robust” subgroups [2]–[5]. One family, the Trachyphylliidae, is trivially distinct and does not merit recognition at the family level. Traditional members of the four remaining families – Acroporidae, Pocilloporidae, Fungiidae, and Dendrophylliidae – cluster together, but the first three of these now contain genera previously assigned to other families. Several genera or groups of genera are so divergent that they will probably need to be assigned to new families. The azooxanthellate Fungiacyathidae was confirmed to be distinctive at the family level (but not closely related to corallimorpharians, see following section). Below we explore these results in somewhat greater detail, comparing the different sources of information (Figs. 1–3), utilizing formal tests of competing phylogenetic hypotheses (Table 1) and exploring implications for needed taxonomic revisions (Table 2, see also Online Supporting Information Table S2).

**Table 1:** Results of hypothesis testing showing p-values for approximately Unbiased (AU), Kishino-Hasegawa (KH), Shimodaira-Hasegawa (SH), and weighted SH (WSH) tests.

	AU	KH	SH	WSH
Acroporidae is monophyletic	**0.000**	**0.000**	**0.000**	**0.000**
Agariciidae is monophyletic	**0.014**	**0.010**	0.887	0.143
Astrocoeniidae is monophyletic	**0.000**	**0.000**	**0.000**	**0.000**
Euphylliidae is monophyletic	**0.000**	**0.000**	**0.000**	**0.000**
Faviidae is monophyletic	**0.000**	**0.000**	**0.000**	**0.000**
Fungiidae is monophyletic	0.215	0.101	0.991	0.804
Meandrinidae is monophyletic	**0.000**	**0.000**	**0.000**	**0.000**
Merulinidae is monophyletic	**0.000**	**0.000**	0.298	**0.001**
Mussidae is monophyletic	**0.000**	**0.000**	**0.028**	**0.000**
Oculinidae is monophyletic	**0.000**	**0.000**	**0.000**	**0.000**
Pectiniidae is monophyletic	**0.000**	**0.000**	0.288	**0.000**
Poritidae is monophyletic	**0.000**	**0.000**	0.087	**0.000**
Siderastreidae is monophyletic	**0.000**	**0.000**	**0.000**	**0.000**
Scleractinia is not monophyletic	0.410	0.234	0.972	0.922

Bold indicates rejection at the 95% confidence level. Because the SH test appears to be too conservative [8], [37] and the KH test is only valid for comparing a priori hypotheses, results of the AU test should be emphasized.

**Table 2:** Tentative groupings of reef-building scleractinian genera based on molecular clades, with total number of species, % data deficient species, and % threatened species for each clade.

Clade	Members	# Spp	% DD	%> = NT/> = VU
II	DEN: *Balanophyllia*, *Duncanopsammia*, *Heteropsammia*, ***Turbinaria***	15	7	71/50
III	POR: *Calathiscus*, ***Goniopora***, ***Porites***, *Poritipora*, *Stylaraea*	87	10	50/28
IV^1^	AGA: ***Pachyseris***	5	0	60/40
V	EUP: *Catalaphyllia*, ***Euphyllia***, *Nemenzophyllia*	22	9	90/60
	MEA: ***Ctenella***, *Gyrosmilia*, *Montigyra*			
	OCU: ***Galaxea***, *Simplastrea*			
	FAV: *Parasimplastrea*			
VI	ACR: ***Acropora***, ***Anacropora***, ***Astreopora***, *Enigmopora*, ***Isopora***, ***Montipora***	285	29	73/51
	POR: ***Alveopora***			
VII	AGA: ***Agaricia***, *Coeloseris*, ***Gardinoseris***, ***Helioseris***, ***Leptoseris***, ***Pavona***	40	7	35/24
VIII	AST: ***Stephanocoenia***	1	0	0/0
IX^2^	SID: ***Pseudosiderastrea***, ***Siderastrea***	6	17	40/20
X	POC: ***Pocillopora***, ***Seriatopora***, ***Stylophora***	45	13	41/26
	AST: ***Madracis***, *Palauastrea*, ***Stylocoeniella***			
XI^2^,^3^	FUN: *Cantharellus*, ***Ctenactis***, ***Fungia***, ***Halomitra***, ***Heliofungia***, ***Herpolitha***, *Lithophylon*, ***Podabacia***, *Polyphyllia*, ***Sandalolitha***, *Zoopilus*	80	9	34/16
	SID: ***Anomastraea***, ***Coscinaraea***, *Craterastrea*, ***Horastrea***, ***Psammocora***			
	FAV: ***Leptastrea***, ***Oulastrea***			
XII	MEA: ***Dendrogyra***, ***Dichocoenia***, ***Eusmilia***, ***Meandrina***	7	29	40/40
XIII	OCU: ***Oculina***, *Schizoculina*	12	50	17/17
	FAV: ***Cladocora***, ***Solenastrea***			
XIV	FAV: ***Plesiastrea***	11	27	75/25
	EUP: ***Physogyra***, ***Plerogyra*** 3			
	MUS: ***Blastomussa***			
XV	FAV: ***Diploastrea***	1	0	100/0
XVI	FAV: ***Montastraea*** (*cavernosa* only)	1	0	0/0
XVII	FAV: *Australogyra*, ***Barabattoia***, ***Caulastraea***, ***Cyphastrea***, ***Echinopora***, *Erythrastrea*, ***Favia*** (Pacific), ***Favites***, ***Goniastrea***, ***Leptoria***, ***Montastraea*** (except *cavernosa*), *Moseleya*, ***Oulophyllia***, ***Platygyra***	135	4	70/22
	MER: *Boninastrea*, ***Hydnophora***, ***Merulina***, *Paraclavarina*, ***Scapophyllia***			
	PEC: ***Mycedium***, ***Pectinia***			
	TRA: ***Trachyphyllia***			
XVIII+XX^4^	MUS: ***Acanthastrea***, ***Micromussa***	15	7	93/43
XIX	MUS: *Australomussa*, ***Cynarina*** 5, ***Lobophyllia***, ***Scolymia*** (Pacific), ***Symphyllia***	34	12	37/20
	PEC: *Echinomorpha*, ***Echinophyllia***, ***Oxypora***			
XXI	FAV: ***Colpophyllia***, ***Diploria***, ***Favia*** (Atlantic), ***Manicina***	21	29	7/7
	MUS: *Isophyllastrea*, ***Isophyllia***, ***Mussa***, ***Mussismillia***, ***Mycetophyllia***, ***Scolymia*** (Atlantic)			

Placement of genera not studied (studied genera in bold) was based on morphological similarity and/or biogeography, and should be regarded as provisional. Names of genera taken from recent analysis of extinction risk [24], which categorized species as data deficient (DD), least concern, or levels of increasing threat [near threatened (NT), vulnerable (VU), endangered, and critically endangered]. In this table, percent of species lacking adequate data, percent of species with moderate (NT or above) and high (VU or above) risk are indicated. Family abbreviations (all capitals) and clade roman numerals are as in Figure 1.

1 Some *Pachyseris* (particularly *P. gemmae* and *P. rugosa*) may be cluster with remainder of Agariciidae in Clade VII.

2 Based also on other analyses [21].

3 Based on unpublished CO1 data.

4 Based on Fig. 2.

5 Includes *Indophyllia* (pers. obs.).

The large family Faviidae Gregory, 1900 is one of the most polyphyletic of all scleractinian families in our analyses, with members scattered throughout the “robust” clade (groups XI, XIII, XIV, XV, XVI, XVII, XXI in Fig. 1D; groups X1-a, XI-b, XIII-a, XIII-b, XIV-b, XVI, XVII-a, XVII-b, XXI in Fig. 2; groups XI, XV, XXI-a in Fig. 3; p<.001 for all four tests of monophyly, Table 1). The type for the family (*Favia fragum*) is in a clade that contains the type for the family Mussidae Ortmann, 1890 (*Mussa angulosa*) (group XXI in Figs. 1D, 2B) [6]; thus the family name Faviidae cannot be retained in the formal taxonomic revisions to come, and the Mussidae will include most of the Atlantic “faviids” with the exception of the polyphyletic genus *Montastraea* (which falls in groups XVI and XVII, Figs. 1D and 2B). Two other families when newly revised will include other former members of the Faviidae - the Oculinidae (*Solenastrea*, *Cladocora*) (group XIII Figs. 1D, 3; group XIII-b but not XIII-a in Fig. 2A) [6] and the Fungiidae (*Leptastrea*) (group XI in Fig. 1D, group XI-b in Fig. 2A). *Oulastrea* appears to be a highly distinctive outgroup to the Fungiidae (group XI in Figs. 1D, 3) that minimally merits recognition at the subfamilial level. *Plesiastrea* is highly divergent, but with no consistently identified close relatives (group XIV in Fig. 1D, group XIV-b in Figs. 2A, 3). *Diploastrea* and *Montastraea cavernosa* are also highly divergent within the robust clade (groups XV and XVI in Fig. 1D, group XVI in Fig. 2B; group XV in Fig. 3). The remaining “faviid” genera form a well defined clade, but it also includes all or some members of three other families: the Merulinidae Verrill, 1866, some of the Pectiniidae Vaughan and Wells, 1943 (*Pectinia*, *Mycedium*; monophyly of Pectiniidae rejected at p<.001 for three of four tests, Table 1), and the Trachyphylliidae Verrill, 1901 (group XVII in Fig. 1D, group XVII-a in Fig. 2B) [6]. Thus this large group could be redescribed under the family name Merulinidae, although it should be noted that even the four original genera of this family do not form a monophyletic assemblage within the larger group (p< = .001 for three of four tests of monophyly, Table 1).

The large family Mussidae is also polyphyletic (p<.001 or .028 for the four tests of monophyly, Table 1). It includes Atlantic and Pacific clades, the first of which, as noted above, will retain the family name and also include most of the Caribbean “faviids” (group XXI in Figs. 1D, 2B) [6]. One Pacific clade clearly includes most of the Pacific “mussids” and some of the pectinids (*Oxypora*, *Echinophyllia*) (group XIX in Figs. 1D, 2B), a grouping that could be recognized at the family or subfamily level. However, the relationships of *Micromussa* and members the genus *Acanthastrea* (and even the monophyly of *Acanthastrea*) are unstable (groups XVIII and XX in Figs. 1D, 2B, 3). *Blastomussa* is highly divergent and its phylogenetic placement remains unstable across analyses (group XIV in Fig. 1D, XIV-a in Fig. 2A).

The family Siderastreidae Vaughan and Wells, 1943 consists of two very distantly related clades (monophyly rejected with p<.001 for all four tests, Table 1). The type genus *Siderastrea* (one of only three monophyletic genera with Pacific and Atlantic species, the others being *Acropora* and *Porites*) has no other close relative in our analyses (group IX in Figs. 1B, 2A, 3), whereas the Pacific genera *Psammocora* and *Coscinaraea* are probably allied to the Fungiidae (group XI in Figs. 1D, 3, but with a different placement as group XI-c in Fig. 2A).

The family Astrocoeniidae Koby, 1890 also contains two highly divergent clades (monophyly rejected at p<.001 for all four tests) – one represented by the Atlantic *Stephanocoenia* (which has no close relatives in our analyses; group VIII in Figs. 1B, 3) and the other represented by *Madracis* and *Stylocoeniella*, which are most closely related to the Pocilloporidae (group X in Figs. 1C, 3). The type genus for the family is a fossil genus (*Astrocoenia*) that appears to be close to *Stephanocoenia* in morphology.

Most other families also have gains and/or losses in their memberships. The Oculinidae Gray, 1847 gains the “faviids” *Cladocora* and *Solenastrea*, as noted above, but loses *Galaxea* to the Euphylliidae Veron, 2000 (group V in Figs. 1B, 2A) (monophyly for Oculinidae rejected at p<.001 for all four tests, Table 1). The family Euphylliidae also gains *Ctenella* from the Meandrinidae Gray, 1847 (group V in Figs. 1B, 2A), and is allied to at least one representative of the genus *Pachyseris* from the family Agariciidae Gray, 1847 as a sister group (group IV in Figs. 1B, 2A) (monophyly of Euphylliidae and Meandrinidae rejected at p<.001 by four of four tests, monophyly of Agariciidae rejected at p<0.02 for two of four tests, Table 1). The former euphylliid *Physogyra* has no close relatives (group XIV in Fig. 1D; group XIV-c in Figs. 2A, 3), although the morphologically similar genus *Plerogyra* appears related based on unpublished data. The Meandrinidae and the Agariciidae are otherwise unaffected by these analyses (although two other Pacific genera remain unstudied in the Meandrinidae, which is otherwise Atlantic in distribution). The Fungiidae Dana, 1846, as noted above, gains *Leptastrea*, *Psammocora*, *Coscinaraea* and *Oulastrea*; although our tests did not reject monophyly for this family based on the mitochondrial data (Table 1), both mitochondrial and nuclear data support this conclusion (group XI in Figs. 1D, 3, group XI-b in Fig. 2). The families Acroporidae Verrill, 1902 and Poritidae Gray, 1842 are largely stable, although the genus *Alveopora* must be transferred from the latter to the former (Fig. 1B, [15]) (monophyly of Acroporidae and Poritidae rejected at p<.001 for four of four and three of four tests respectively, Table 1). The family Pocilloporidae Gray, 1842 retains all its conventionally assigned genera (and thus remains monophyletic), but gains new members via a sister clade, as noted above, that contains *Stylocoeniella* and *Madracis* (group X in Figs. 1C, 3). Only the Dendrophylliidae Gray, 1847 remains unchanged in composition and without new close relatives.

### Status of the Order Corallimorpharia

Medina et al. [8] suggested that corallimorpharians are scleractinian corals which have secondarily lost the skeleton. Our data do not support this hypothesis. Independent analysis of ribosomal DNA and tubulin data both strongly indicate that Corallimorpharia lies outside Scleractinia (Figs. 2A, 3). Mitochondrial data are more equivocal, in that some trees (e.g. maximum parsimony analysis, see methods) lend support to a link between the complex corals and the corallimorpharians, but our maximum likelihood/Bayesian analyses of the full taxonomic data set using *cox1* and *cob* place the corallimorpharians outside the scleractinians (see also [9]) (although the hypothesis of non-monophyly for the Scleractinia was not rejected by the mitochondrial data, Table 1).

## Discussion

Genetic analyses have transformed our understanding of evolutionary relationships over the last decades. In some cases such as families of angiosperms, molecular data have provided important refinements or helped to resolve long-standing controversies, but left many traditional taxonomic groupings intact [16]. In other cases such as sponges, molecular data have overturned much traditional taxonomy and highlighted many previously unanticipated groupings [17].

Although taxonomic problems for corals at the species level have been compared to species ambiguities in angiosperms [18], at higher taxonomic levels the situation for corals resembles that of many sponges, which also lack organ systems and typically a diversity of complex macro-morphological structures. Almost all the families we analyzed either gained or lost members, and in some cases the changes are very substantial. Studies of families dominated by azooxanthellate corals also indicate extensive polyphyly [4], [12], [13]. Most strikingly, at least seven families (five analyzed here plus the Caryophylliidae and the Guyniidae [7], [13]) have conventionally defined members in both the complex and robust clades, which all analyses indicate are highly divergent genetically [2]–[5], [10].

Although more work remains to be done, our conclusions are robust to a number of possible problems. First, nuclear and mitochondrial data sets give broadly similar results. We tested for family monophyly (Table 1) using the largest data set (*cox1*+*cob*, Fig. 1), and in every case where monophyly was rejected using formal tests (Table 1), the conclusion is also supported by non-monphyletic topologies in the tubulin data set (Fig. 2), the r-DNA data set (Fig. 3), or both. This makes mitochondrial pseudogenes a highly unlikely explanation for the extent of non-monophyly of scleractinian families in our mitochondrial analyses. Second, although geographic sampling is limited (that is, most species were collected from a single location), where samples from multiple locations were available (e.g. *Montastraea cavernosa*, *Favia fragum*, and *Scolymia cubensis* from Brazil and Panama; *Plesiastrea versipora* from Palau and Japan, *Siderastrea savignyana* from Taiwan and Oman; Online Supporting Information Table S1), sequences were either identical, sister taxa, or grouped with all other members of the genus on the mitochondrial tree (Fig. 1). Hybridization is unlikely to contribute to patterns at the level of families or above since it has not been reported between members of different genera.

Given these results, traditional morphological characters must be plagued by convergence. Several examples emerged from our previous study [6] and there are numerous others. For example, fenestrate septa are found in both complex corals (Poritidae, Siderastreidae) and robust corals (Fungiidae) and may not be homologous in the two cases. Similarly, synapticulae are found in most but not all complex corals (lacking in the Astrocoeniidae and Euphylliidae) and are absent in most but not all robust corals (present in the Fungiidae and its allies). In addition, some taxa have been included in families because of their overall similar appearance, despite having several characters atypical of the families to which they are currently assigned, including characters noted by previous authors (e.g. *Oulastrea* and *Madracis* [19], *Leptastrea* and *Plesiastrea* [20]). Other examples of morphological support for initially surprising molecular results are summarized in Online Supporting Information (Table S2). More comprehensive formal morphological analyses in light of emerging molecular data (e.g. [21], [22]) are clearly needed.

The results reported here also strengthen the conclusion of Fukami and colleagues [6] that the distinctiveness of the Atlantic scleractinian taxa has been underappreciated. Several families appear to be now largely or exclusively Atlantic: the newly defined Mussidae, the Meandrinidae, the Oculinidae, and the divergent taxon *Montastraea cavernosa*. *Stephanocoenia* may represent another distinctive Atlantic clade, whose modern members consist of only one genus, but the Pacific astrocoeniid genus *Palauastrea* must be analyzed to confirm this.

The last decade has brought much change to our understanding of scleractinian relationships, but much still needs to be done. Some zooxanthellate genera remain to be analyzed (Online Supporting Information Table S2, plus many azooxanthellates), and firm conclusions about biogeographic distributions and the prevalence of families containing single genera are thus premature. Other genera require additional work either because they are so divergent that phylogenetic analyses are difficult (e.g. long branch attraction [15]) or because the genus itself contains highly divergent species so that conclusions depend on which species are studied. An example of the former is found in the uncertain phylogenetic placement of three small divergent genera [*Blastomussa* (2 spp), *Physogyra* (1 spp), and *Plesiastrea* (2 spp)]. Examples of the latter are several genera that appear to have members which are highly divergent even within ocean basins, for example *Acanthastrea* (*A. hillae* and *A. echinata* are divergent in Fig. 1D but not in Fig. 2B) and *Pachyseris* [one species of which is close to the Euphylliidae (Figs. 1B, 2A) whereas at least some of the others appear to be good members of the Agariciidae (Hoeksema, unpubl.)]. The need for accurate species identifications (often a challenge in corals) and skeletal vouchers to back up identifications is particularly acute in such cases. Nevertheless, an outline of the family tree based on a diverse array of molecular markers does appear to be emerging for the Scleractinia. With it comes the opportunity to redefine families based on morphological characters, which can then be traced through the fossil record.

Our results also call into question the hypothesis that the corallimorpharians are “naked” corals that have secondarily lost their skeleton. Three independent analyses yield trees that support the monophyly of the Scleractinia within the Hexacorallia. The mitochondrial tree was rooted by taxa representing the Zoanthidea, Actiniaria and Antipatharia; the tubulin tree was rooted by a member of the Antipatharia, and the rDNA tree was rooted by members of the Actiniaria and the Zoanthidea, as well as by a member of the Octocorallia, so issues of rooting are unlikely to be responsible (see [9] for an extensive analysis of the mitochondrial genome of these groups that also concludes that the Scleractinia are monophyletic). Moreover, Medina et al. [8] report strong gene order differences between the Corallimorpharia and the Scleractinia, which would be consistent with the idea that the Scleractinia are a monophyletic group that does not include the Corallimorpharia. Thus while the traditional relationships within the Scleractinia are very poorly supported, the group itself appears to be derived from a single evolutionary lineage.

Finally, accurate understanding of evolutionary relationships has implications for ecology and conservation. For example, the conclusion that members of the Faviidae are resistant to environmental stress because they are over-represented in areas of low diversity [23] needs to be reexamined in light of the fact that “faviids” appear in at least seven of the 21 molecular clades (Fig. 1, Table 2). Our reanalysis of extinction risks for these clades using the recently published listing for all reef-building corals [24] highlights the vulnerabilities of clades II, V, VI, XV, and XVIII+XX, and the lack of adequate information for clades VI, XII, XIII, XIV, and XXI (Table 2). Our ability to protect deep lineages most at risk [25] depends on knowing what these lineages are.

## Materials and Methods

### Collections and DNA extraction

We collected 15 cm×15 cm samples from the following locations: Bocas del Toro, Republic of Panama (Caribbean coast); Carrie Bow Cay, Belize; Togian Island, Indonesia; Aka Island, Okinawa and Shirahama, Wakayama, Japan; Yeiliu, Suao, Kenting National Park and Penghu Island, Taiwan (see Supporting Information Table S1). Total DNA extraction method is as described in Fukami et al. [6].

### DNA sequencing

The mitochondrial *cox1* gene was amplified by polymerase chain reaction (PCR) with previously reported primers MCOIF and MCOIR [6] for most “robust” corals. For a few “robust” coral species (especially pocilloporids), “complex” corals, corallimorpharians and other hexacorallians, combinations (e.g. primer pair of AcMCOIF and SeaMCOIR for PCR) of the following newly designed primers were used: AcMCOIF (5′-GAC ATG GCT ATT TTT AGC CT-3′), SeaMCOIF (5′-CTA CTA ATC ATA AAG ATA TCG G-3′), AcMCOIR (5′-AAG CAT AGG AGT GTC GTC TAA TC-3′), SeaMCOIR (5′-CAA AGT CAG AGT ATC GTC TTG G-3′).

Mitochondrial *cob* was amplified by PCR with previously reported primers MCytbF and MCytbR for “robust” corals [6], and with newly designed primers AcCytbF (5′-GCC GTC TCC TTC AAA TAT AAG-3′) or MCytbF, and AcCytbR (5′-AAA AGG CTC TTC TAC AAC-3′) for “complex” corals and other hexacorallians except for the three sea anemone species. For the sea anemones, SeaCytbF (5′-GTG GAA CTT CGG TTC TTT ATT-3′) and SeaCytbR (5′-ATA CAG AGG CTA ATT GTC C-3′) were used for PCR.

The basic protocol for mitochondrial gene amplifications was 94°C for 120 s, followed by 30 cycles at 94°C for 45 s, 55°C for 45 s and 72°C for 90 s, ending with a final phase of 72°C for 5 min. In the case of amplification failure, the following protocol was used: 94°C for 120 s, followed by first 10 cycles at 94°C for 45 s, 45°C for 45 s and 72°C for 90 s, and next 20 cycles at 94°C for 45 s, 45°C for 45 s and 72°C for 90 s, ending with a final phase of 72°C for 5 min. PCR products were directly sequenced as described previously [6].

The ß-tubulin gene (intron and exon regions) was amplified, cloned, and sequenced following the methods described previously [6].

The ribosomal DNA (rDNA) segment containing the 3′-end of 18S, internal transcribed spacers, 5.8S, and the 5′-end of 28S was amplified, cloned, and sequenced following the methods described elsewhere [15], [26].

DNA sequence accession numbers of all genes analyzed here (AY722757, AY722761, AY722775, AY722781, AY722785, AY722793-6, AB441193-AB441421) are listed in the Table S1 (Online Supporting Information).

### DNA phylogenetic analyses

For mitochondrial DNA, we conducted phylogenetic analyses on the dataset [total 1383 bp, combined *cox1* (607 bp) and *cob* (776 bp) without gaps; 666 parsimony-informative sites] by searching for optimal topologies, as measured by the criteria maximum likelihood (ML) and parsimony (MP) methods, as well as by taking a Bayesian approach. For the ML and Bayesian analyses, we assumed a model of nucleotide evolution obtained by using the Akaike Information Criterion (AIC) as implemented in ModelTest [27]. The most appropriate model of nucleotide evolution for the mitochondrial datasets was the GTR with gamma (G) and invariant (I) parameters (GTR+I+G). The software GARLI [28] was used to search for optimal ML topologies (10 replicate searches started with random trees) and to conduct bootstrap analyses (200 replicates). The program TNT [29] was employed to conduct searches for optimal MP trees (10 replicate tree bisection-reconnection searches with taxa added randomly) and to assess node support with re-sampling methods (1000 replicate bootstrap and jackknife searches). MrBayes [30] was used to conduct Bayesian analyses (with default priors and nruns = 3, samplefreq = 1000, nchains = 8). The number of generations and burnin in millions for the mitochondrial datasets were 8 and 4, respectively. As judged by the potential scale reduction factor (PSRF), analyses converged.

Topologies from ML and Bayesian analyses were quite similar. Topology from MP analysis was also largely similar to others obtained, and most nodes in the MP tree that received bootstrap support appeared as part of the Bayesian analysis with a Bayesian posterior probability greater than 0.95. However, topology of the MP tree differed in one major point, namely the phylogenetic position of the corallimorpharians. The MP tree showed a link between the corallimorpharians and the “complex” corals (as in [8]), whereas ML and Bayesian trees showed corallimorpharians to be outside the scleractinian corals. In this paper, we show the topology of Bayesian analysis with ML bootstrap values and a Bayesian posterior probability. Three species in the order Actiniaria (*Anemonia* sp., *Stichodactyla* sp., *Metridium senile* [31]), one species in the order Anthipatharia (*Cirripathes* sp.) and one species in the order Zoanthidea (*Zoanthus* sp.) were used for outgroups in the mitochondrial analyses (Fig. 1).

In order to further assess various phylogenetic hypotheses not found in our best trees, we tested whether our mitochondrial data significantly contradict these suboptimal hypotheses. Using GARLI [28], we conducted ten replicate searches for most likely topologies constrained to conform to each of 14 hypotheses (Table 1). The program CONSEL [32] was then used to calculate p-values for several tests – including the Approximately Unbiased (AU) [33], Kishino-Hasegawa (KH) [34], Shimodaira-Hasegawa (SH); [35], and the weighted SH (WSH) [30] tests – aimed at measuring how strongly the various hypotheses are contradicted by the data. Because the SH test is only valid when numerous plausible trees are being compared, all resulting trees from the constrained and unconstrained searches were compared.

For ß-tubulin, previously published DNA sequences [6] were retrieved from the database and added to newly obtained sequences for phylogenetic analysis. For the phylogenetic tree construction, only the exon region of ß-tubulin was utilized, since the intron region is too diverse to produce reliable alignments above the generic level [6]. When all taxa were used for the phylogenetic analyses, Bayesian probability and ML bootstrap values were too low to support the main branches due to the limited number of informative sites, probably because of the phylogenetically distant relationships among species. Therefore, data were separated into two data sets (Tub1 for the “complex” group plus some “robust” corals; Tub2 for most robust corals). The aligned ß-tubulin fragment was 443 bp in length, with 123 and 87 phylogenetically informative sites for Tub1 and Tub2, respectively. As described for the mitochondrial analyses, the most appropriate models of nucleotide evolution for the ML and Bayesian analyses were determined to be HKY+I+G for Tub1 and TrN+I+G for Tub2. PAUP 4.0b10 [36] was used for both Tub1 and Tub2 to reconstruct the ML tree. The robustness of the phylogenies was assessed using the 300 bootstrap option. Bayesian trees for Tub1 and Tub2 were constructed as described for mitochondrial analyses. Four simultaneous Markov chains were run for 1,200,000 (Tub1) or 2,000,000 (Tub2) generations; trees were sampled every 100 generations, with 300,000 (Tub1) or 500,000 (Tub2) initial trees discarded as burn-in, based on visual inspection. In this paper, we show the topology of the Bayesian analysis with ML bootstrap values and Bayesian posterior probabilities. One species in the Antipatharia (*Cirripathes* sp.) was used as an outgroup to the Scleractinia and Corallimorpharia.

For ribosomal DNA, previously published DNA sequences [15], [26] were retrieved from the database and added to new sequences for phylogenetic analysis. For the phylogenetic tree construction, only the 3′-end of 18S, 5.8S, and the 5′-end of 28S rDNA were utilized, since the internal transcribed spacers are too divergent to produce reliable alignments above the generic level [15], [21]. The Acroporidae were also excluded from the analysis due to their extremely high rates of rDNA evolution compared to other scleractinian corals [15]. The aligned rDNA fragment was 554 bp in length, and 70 sites were phylogenetically informative. As described for the mitochondrial analyses, the most appropriate model of nucleotide evolution for the ML and Bayesian analyses was determined to be the TIM+I+G model. PAUP 4.0b10 was used to reconstruct the ML tree. The robustness of the phylogenies was assessed using the 1000 bootstrap option. The Bayesian tree was constructed as described for the mitochondrial analyses. Five simultaneous Markov chains were run for 1,000,000 generations with trees sampled every 10 generations, with 50,000 initial trees discarded as burn-in, based on visual inspection. In this paper, we show the topology of the Bayesian analysis with ML bootstrap values and Bayesian posterior probabilities. One member of the Octocorallia (*Heliopora coerulea*), one member of the Zoanthidea (*Sphenopus marsupialis*) and one member of the Actiniaria (*Condylactis* sp.) were used as outgroups to the Scleractinia and Corallimorpharia.

## Supporting Information

Table S1 Species lists, localities, and accession numbers.
(0.42 MB DOC)

Table S2 Summary of possible changes to current taxonomy of reef-building corals [(1) for most corals, (2) for Fungiidae] and evidence supporting those changes. We list provisional placement based on mitochondrial data (from cox1 and cob from Fig. 1 unless otherwise noted); sources of additional evidence that supports the mitochondrial data are indicated in footnotes [some of these data also appeared in Fukami et al. (3)]. Note that not all members of speciose genera have been examined; in some cases these genera may ultimately be distributed among families, and we list species names where we know that different species have substantially different phylogenetic placements. This table suggests an outline for a revised taxonomy but does not represent a formal taxonomic revision. Families that are exclusively or almost exclusively azooxanthellate (Rhizangiidae, Caryophylliidae) are not included. In addition, the genera Blastomussa, Micromussa, Physogyra, and Plesiastrea are not included in lists of new affiliations because mitochondrial and nuclear data provide no consistent indication of likely close relatives.
(0.10 MB DOC)

## References

[1] Shearer TL, van Oppen MJH, Romano SL, Wörheide G (2002). Slow mitochondrial DNA sequence evolution in the Anthozoa (Cnidaria).. Mol Ecol.

[2] Romano SL, Palumbi SR (1996). Evolution of scleractinian corals inferred from molecular systematics.. Science.

[3] Romano SL, Palumbi SR (1997). Molecular evolution of a portion of the mitochondrial 16S ribosomal gene region in scleractinian corals.. J Mol Evol.

[4] Romano SL, Cairns SD (2000). Molecular phylogenetic hypotheses for the evolution of scleractinian corals.. Bull Mar Sci.

[5] Chen CA, Wallace CC, Wolstenholme J (2002). Analysis of mitochondrial 12S RNA gene supports a two-clade hypothesis of the evolutionary history of scleractinian corals.. Mol Phyl Evol.

[6] Fukami H, Budd AF, Paulay G, Sole-Cava A, Chen CA (2004). Conventional taxonomy obscures deep divergence between Pacific and Atlantic corals.. Nature.

[7] Kerr AM (2005). Molecular and morphological supertree of stony corals (Anthozoa: Scleractinia) using matrix representation parsimony.. Biol Rev.

[8] Medina M, Collins AG, Takaoka TL, Kuehl JV, Boore JL (2006). Naked corals: Skeleton loss in Scleractinia.. Proc Natl Acad Sci U S A.

[9] Brugler MR, France SC (2007). The complete mitochondrial genome of the black coral *Chrysopathes formosa* (Cnidaria:Anthozoa:Antipatharia) supports classification of antipatharians within the subclass Hexacorallia.. Mol Phyl Evol.

[10] Veron JEN, Odorico DM, Chen CA, Miller DJ (1996). Reassessing evolutionary relationships of scleractinian corals.. Coral Reefs.

[11] Daly M, Fautin DG, Cappola VA (2003). Systematics of the Hexacorallia (Cnidaria: Anthozoa).. Zool J Linn Soc.

[12] Le Goff-Vitry MC, Rogers AD, Baglow D (2004). A deep-sea slant on the molecular phylogeny of the Scleractinia.. Mol Phyl Evol.

[13] Cuif JP, Lecointre G, Perrin C, Tillier A, Tillier S (2003). Patterns of septal biomineralization in Scleractinia compared with their 28S rRNA phylogeny: a dual approach for a new taxonomic framework.. Zool Scri.

[14] den Hartog JC (1980). Caribbean shallow water Corallimorpharia.. Zool Verhand Leiden.

[15] Wei WV, Wallace CC, Dai CF, Moothien Pillay RK, Chen CA (2006). Analyses of the ribosomal internal transcribed spacers (ITS) and the 5.8S gene indicate that extremely high rDNA heterogeneity is a unique feature in the scleractinian coral genus *Acropora* (Scleractinia; Acroporidae).. Zool Stud.

[16] Chace MW, Fay MF, Savolainen V (2000). Higher-level classification in the angiosperms: new insights from the perspective of DNA sequence data.. Taxon.

[17] Boury-Esnault N (2006). Systematics and evolution of Demospongiae.. Can J Zool.

[18] Willis BL, van Oppen MJH, Miller DJ, Vollmer SV, Ayre DJ (2006). The role of hybridization in the evolution of reef corals.. Ann Rev Ecol Evol Syst.

[19] Vaughan TW, Wells JW (1943). Revision of the suborders, families and genera of the Scleratinia.. Geol Soc Am Spec Pap.

[20] Alloiteau J (1957). Contribution a la systématique des madréporaires fossiles.. CNRS, Paris.

[21] Benzoni F, Stefani F, Stolarski J, Pichon M, Mitta G (2007). Debating phylogenetic relationships of the scleractinian *Psammocora*: molecular and morphological evidences.. Contr Zool.

[22] Budd AF, Stolarksi J (2008). Searching for new morphological characters in the systematics of scleractinian reef corals: comparison of septal teeth and granules between Atlantic and Pacific Mussidae.. Acta Zool.

[23] Bellwood DR, Hughes TP (2001). Regional-scale assembly rules and biodiversity of coral reefs.. Science.

[24] Carpenter KE, Abrar M, Aeby G, Aronson RB, Banks S (2008). One third of reef-building corals face elevated extinction risk from climate change and local impacts.. Science.

[25] Mace GM, Gittleman JL, Purvis A (2003). Preserving the tree of life.. Science.

[26] Chen CA, Chang CC, Wei NV, Chen CH, Lein IT (2004). Secondary structure and phylogenetic utility of the ribosomal internal transcribed spacer 2 in the scleractinian corals.. Zool Stud.

[27] Posada D, Crandall KA (1998). Modeltest: Testing the model of DNA substitution.. Bioinformatics.

[28] Zwickl DJ (2006). Genetic algorithm approaches for the phylogenetic analysis of large biological sequence datasets under the maximum likelihood criterion, ver. 0.95.. http://www.bio.utexas.edu/faculty/antisense/garli/Garli.html.

[29] Goloboff P, Farris S, Nixon K (2000). TNT:Tree analysis using New Technology (BETA), ver. 1.0.

[30] Ronquist R, Huelsenbeck JP (2003). MrBayes 3: Bayesian phylogenetic inference under mixed models.. Bioinformatics.

[31] Beagley CT, Okimoto R, Wolstenholme DR (1998). The mitochondrial genome of the sea anemone *Metridium senile* (Cnidaria): introns, a paucity of tRNA genes, and a near-standard genetic code.. Genetics.

[32] Shimodaira H, Hasegawa M (2001). CONSEL: For assessing the confidence of phylogenetic tree selection.. Bioinformatics.

[33] Shimodaira H (2002). An approximately unbiased test of phylogenetic tree selection.. Syst Biol.

[34] Kishino H, Hasegawa M (1989). Evaluation of the maximum-likelihood estimate of the evolutionary tree topologies from DNA sequence data, and the branching order in Hominoidea.. J Mol Evol.

[35] Shimodaira H, Hasegawa M (1999). Multiple comparisons of log-likelihoods with applications to phylogenetic inference.. Mol Biol Evol.

[36] Swofford DL (2002). PAUP*:Phylogenetic Analysis Using Parsimony (and Other Methods), version 4.0b10.

[37] Buckley TR (2002). Model misspecification and probabilistic tests of topology: Evidence from empirical data sets.. Syst Biol.

