# Supplementary material for: Mitochondrial and Nuclear Genes Suggest that Stony Corals Are Monophyletic but Most Families of Stony Corals Are Not (Order Scleractinia, Class Anthozoa, Phylum Cnidaria)
Source: PLoS One. 2008 Sep 16;3(9):e3222. doi: 10.1371/journal.pone.0003222 (PMC2528942; doi:10.1371/journal.pone.0003222)
Supplement: Table S1 — Species lists, localities, and accession numbers. (0.42 MB DOC) [file pone.0003222.s001.doc]

Table S1. Species lists, localities, and accession numbers.

| Traditional taxonomy | | | Collection locality | Location of voucher | Accession Number | | |  |
| --- | --- | --- | --- | --- | --- | --- | --- | --- |
| Family (Scleractinia) or Order (Outgroups) | *Genus* | *species* |  |  | COI | Cytb | ß-tubulin | rDNA |
| Faviidae | *Barabattoia* | *amicorum* | Aka Island, Okinawa, Japan | SMBL | AB441193 | AB441278 | - | - |
|  | *Caulastraea* | *furcata* | Aka Island, Okinawa, Japan | SMBL | AB117274 [1] | AB117355 [1] | AB118408-9 [1] | - |
|  | *Cladocora* | *arbuscula* | Bocas del Toro, Panama | UI | AB117292 [1] | AB117377-8 [1] | AB118426-7 [1] | - |
|  | *Colpophyllia* | *natans* | Bocas del Toro, Panama | UI | AB117228 [1] | AB117306 [1] | AB118348-9 [1] | - |
|  | *Cyphastrea* | *serailia* | Aka Island, Okinawa, Japan | SMBL | AB117257-8 [1] | AB117334-5 [1] | AB118383 [1] | - |
|  |  | *chalcidicum* | Yeiliu, Taiwan | BRCAS | AB117259 [1] | AB117336 [1] | AB118384-5 [1] | - |
|  | *Diploria* | *strigosa* | Bocas del Toro, Panama | UI | AB117225 [1] | AB117302 [1] | AB118345 [1] | AB441396 |
|  |  | *clivosa* | Bocas del Toro, Panama | UI | AB117226 [1] | AB117304 [1] | AB118346 [1] | - |
|  |  | *labyrinthiformis* | Bocas del Toro, Panama | UI | AB117224 [1] | AB117302 [1] | AB118343-4 [1] | - |
|  | *Diploastrea* | *heliopora* | Aka Island, Okinawa, Japan | SMBL | AB117290 [1] | AB117375 [1] | - | - |
|  |  | *heliopora* | Taiwan | BRCAS | - | - | - | AB441397 |
|  | *Echinopora* | *pacificus* | Aka Island, Okinawa, Japan | SMBL | AB117261-2 [1] | AB117340-1 [1] | AB118386-7 [1] | - |
|  |  | *gemmacea* | Aka Island, Okinawa, Japan | SMBL | AB117263 [1] | AB117342 [1] | AB118388-9 [1] | - |
|  | *Favia* | *pallida* | Aka Island, Okinawa, Japan | SMBL | AB117265-6 [1] | AB117344-5 [1] | AB118391-2 [1] | - |
|  |  | *speciosa* | Shirahama, Wakayama, Japan | SMBL | AB441194 | AB441279 | - | - |
|  |  | *favus* | Aka Island, Okinawa, Japan | SMBL | AB117267 [1] | AB117346 [1] | AB118393 [1] | - |
|  |  | *stelligera* | Aka Island, Okinawa, Japan | SMBL | AB117264 [1] | AB117343 [1] | AB118390 [1] | - |
|  |  | *leptophylla* | Abrolhos, Brazil | FURJ | AB117229-30 [1] | AB117307 [1] | AB118350 [1] | - |
|  |  | *fragum* | Bocas del Toro, Panama | UI | AB117222 [1] | AB117300 [1] | AB118341 [1] | - |
|  |  | *fragum* | Abrolhos, Brazil | FURJ | AB117223 [1] | AB117301 [1] | AB118342 [1] | - |
|  | *Favites* | *halicora* | Aka Island, Okinawa, Japan | SMBL | AB117268 [1] | AB117347-8 [1] | AB118398 [1] | - |
|  |  | *chinensis* | Aka Island, Okinawa, Japan | SMBL | AB117269 [1] | AB117349 [1] | - | - |
|  |  | unidentified*^1^ | Aka Island, Okinawa, Japan | SMBL | AB117271 [1] | AB117351 [1] | AB118395 [1] | - |
|  | *Goniastrea* | *pectinata* | Aka Island, Okinawa, Japan | SMBL | AB117270 [1] | AB117350 [1] | AB118394 [1] | - |
|  |  | *deformis* | Shirahama, Wakayama, Japan | SMBL | AB441195 | AB441280 | - | - |
|  |  | *aspera* | Penghu Island, Taiwan | BRCAS | - | - | - | AY722761 |
|  | *Leptastrea* | *pruinosa* | Yeiliu, Taiwan | BRCAS | AB441196 | AB441281 | AB441363 | - |
|  | *Leptoria* | *irregularis* | Aka Island, Okinawa, Japan | SMBL | AB117272 [1] | AB117352-3 [1] | AB118396 [1] | - |
|  |  | *phrygia* | Aka Island, Okinawa, Japan | SMBL | AB117273 [1] | AB117354 [1] | AB118397 [1] | - |
|  | *Manicina* | *areolata* | Bocas del Toro, Panama | UI | AB117227 [1] | AB117305 [1] | AB118347 [1] | - |
|  | *Montastraea* | *curta* | Aka Island, Okinawa, Japan | SMBL | AB117278 [1] | AB117359 [1] | AB118399-400 [1] | AY722775 |
|  |  | *annularis* complex | Bocas del Toro, Panama | UI | AB117260 [1] | AB117337-9 [1] | U60604 [2] | - |
|  |  | *magnistellata* | Aka Island, Okinawa, Japan | SMBL | AB117279 [1] | AB117360 [1] | AB118401-2 [1] | - |
|  |  | *cavernosa* | Bocas del Toro, Panama | UI | AB117288 [1] | AB117373 [1] | AB118423-4 [1] | - |
|  |  | *cavernosa* | Abrolhos, Brazil | FURJ | AB117289 [1] | AB117374 [1] | AB118425 [1] | - |
|  |  | *valenciennesi* | Aka Island, kinawa, Japan | SMBL | AB117280 [1] | AB117361 [1] | AB118403-4 [1] | - |
|  | *Oulastrea* | *crispata* | Penghu Island, Taiwan | BRCAS | AB441197 | AB441282 | AB441364 | AY722781 |
|  | *Oulophyllia* | *crispa* | Palau | FMRH | AB117275-6 [1] | AB117356-7 [1] | AB118410 [1] | - |
|  |  | *bennettae* | Palau | FMRH | AB117277 [1] | AB117358 [1] | AB118411 [1] | - |
|  | *Platygyra* | *daedalea* | Aka Island, Okinawa, Japan | SMBL | AB117281 [1] | AB117362 [1] | AB118405 [1] | - |
|  |  | *lamellina* | Aka Island, Okinawa, Japan | SMBL | AB117282 [1] | AB117363 [1] | AB118406-7 [1] | - |
|  | *Plesiastrea* | *versipora* | Palau | FMRH | AB289561 [3] | AB289566 [3] | - | - |
|  |  | *versipora* | Shirahama, Wakayama, Japan | SMBL | same as AB289561 | same as AB289566 | AB441365 | AB441398 |
|  | *Solenastrea* | *bournoni* | Bocas del Toro, Panama | UI | AB117291 [1] | AB117376 [1] | AB441366-7 | AB441399 |
| Trachyphylliidae | *Trachyphyllia* | *geoffroyi* | unknown | FMRH | AB117287 [1] | AB117372 [1] | AB118421-2 [1] | - |
| Merulinidae | *Scapophyllia* | *cylindrica* | Wanlitung, Taiwan | BRCAS | AB441198 | AB441283 | - | - |
|  | *Merulina* | *ampliata* | Aka Island, Okinawa, Japan | SMBL | AB117283 [1] | AB117368 [1] | AB118416 [1] | - |
|  |  | *scabricula* | Aka Island, Okinawa, Japan | SMBL | AB117284 [1] | AB117369 [1] | AB118417 [1] | - |
|  | *Hydnophora* | *exesa* | Aka Island, Okinawa, Japan | SMBL | AB117285 [1] | AB117370 [1] | AB118418-9 [1] | - |
|  |  | *grandis* | Aka Island, Okinawa, Japan | SMBL | AB117286 [1] | AB117371 [1] | AB118420 [1] | - |
| Pectiniidae | *Echinophyllia* | *echinoporoides* | Palau | FMRH | AB117254 [1] | AB117331 [1] | AB118379-80 [1] | - |
|  |  | *aspera* | Palau | FMRH | AB117252 [1] | AB117329 [1] | AB118377 [1] | AB441400 |
|  |  | *orpheensis* | Palau | FMRH | AB117253 [1] | AB117330 [1] | AB118378 [1] | - |
|  |  | unidentified | Palau | FMRH | AB117256^†^[1] | AB117333^†^[1] | - | - |
|  | *Pectinia* | *alcicornis* | Palau | FMRH | AB117385 [1] | AB117364 [1] | AB118412 [1] | - |
|  |  | *paeonia* | Palau | FMRH | AB117386 [1] | AB117365 [1] | AB118413 [1] | - |
|  | *Mycedium* | *elephantotus* | Palau | FMRH | AB117387-8 [1] | AB117366-7 [1] | AB118414-5 [1] | - |
|  | *Oxypora* | *lacera* | Palau | FMRH | AB117255 [1] | AB117332 [1] | AB118381-2 [1] | - |
| Mussidae | *Lobophyllia* | *corymbosa* | Aka Island, Okinawa, Japan | SMBL | AB117241 [1] | AB117318 [1] | AB118363 [1] | - |
|  |  | *hemprichii* | Aka Island, Okinawa, Japan | SMBL | AB117240 [1] | AB117317 [1] | AB118362 [1] | - |
|  |  | *pachysepta* | Aka Island, Okinawa, Japan | SMBL | AB117242 [1] | AB117319 [1] | AB118364 [1] | - |
|  | *Symphyllia* | *agaricia* | Aka Island, Okinawa, Japan | SMBL | AB117243 [1] | AB117320 [1] | AB118365-6 [1] | - |
|  |  | *radians* | Aka Island, Okinawa, Japan | SMBL | AB117245 [1] | AB117322 [1] | AB118369 [1] | - |
|  |  | *recta* | Aka Island, Okinawa, Japan | FMRH | AB117244 [1] | AB117321 [1] | AB118367-8 [1] | - |
|  | *Scolymia* | Unidentified | Palau | FMRH | AB117248 [1] | AB117325 [1] | AB118374-5 [1] | - |
|  |  | *vitiensis* | Palau | FMRH | AB117247 [1] | AB117324 [1] | AB118372-3 [1] | - |
|  |  | *cubensis* | Bocas del Toro, Panama | UI | AB117236 [1] | AB117313 [1] | AB118358 [1] | - |
|  |  | *cubensis* | Abrolhos, Brazil | FURJ | AB117237 [1] | AB1173134 [1] | AB1183589 [1] | - |
|  | *Cynarina* | *lacrymalis* | unknown | FMRH | AB117246 [1] | AB117323 [1] | AB118370-1 [1] | - |
|  | *Acanthastrea* | *echinata* | Aka Island, Okinawa, Japan | SMBL | AB117249-50 [1] | AB117326-7 [1] | AB118376 [1] | AB441401 |
|  |  | *rotundoflora* | Palau | FMRH | AB117251 [1] | AB117328 [1] | - | - |
|  |  | *hillae* | Wakayama, Japan | SMBL | AB441199 | AB441284 | AB441368-9 | - |
|  | *Mycetophyllia* | *danaana* | Bocas del Toro, Panama | UI | AB1173234 [1] | AB117311 [1] | AB118356 [1] | - |
|  |  | *aliciae* | Bocas del Toro, Panama | UI | AB117235 [1] | AB117312 [1] | AB118357 [1] | - |
|  | *Isophyllia* | *sinuosa* | Bocas del Toro, Panama | UI | AB117238 [1] | AB117315 [1] | AB118360 [1] | - |
|  | *Mussa* | *angulosa* | Bocas del Toro, Panama | UI | AB117239 [1] | AB117316 [1] | AB118361 [1] | AB441402 |
|  | *Mussismilia* | *braziliensis* | Abrolhos, Brazil | FURJ | AB117231 [1] | AB117309 [1] | AB118351 [1] | - |
|  |  | *harttii* | Abrolhos, Brazil | FURJ | AB117232 [1] | AB117308 [1] | AB118352-3 [1] | - |
|  |  | *hispida* | Abrolhos, Brazil | FURJ | AB117233 [1] | AB117310 [1] | AB118354-5 [1] | - |
|  | *Micromussa* | *amakusensis* | Shirahama, Wakayama, Japan | SMBL | AB441200 | AB441285 | AB441370-1 | AB441403 |
|  | *Blastomussa* | *wellsi* | Palau | FMRH | AB289563 [3] | AB289565 [3] | - | - |
|  |  | *wellsi* | Kaohsiung, Taiwan | BRCAS | - | - | AB441372-3 | - |
| Oculinidae | *Oculina* | *diffusa* | Bocas del Toro, Panama | UI | AB117293 [1] | AB117379 [1] | AB118428-9 [1] | AB441404 |
|  | *Galaxea* | *fascicularis* | Aka Island, Okinawa, Japan | SMBL | AB441201-2 | AB441286-7 | AB441374-5 | - |
| Euphylliidae | *Physogyra* | *lichtensteini* | Palau | FMRH | AB289562 [3] | AB289564 [3] | - | - |
|  |  | *lichtensteini* | Kaohsiung, Taiwan | no specimen | - | - | AB441376 | AB441405 |
|  | *Euphyllia* | *divisa* | Kenting, Taiwan | BRCAS | AB441203 | AB441288 | - | - |
|  |  | *glabrescens* | Kenting, Taiwan | BRCAS | AB441206 | AB441291 | AB441377 | - |
|  |  | *glabrescens* | Aka Island, Okinawa, Japan | SMBL | AB441207 | AB441292 | - | - |
|  |  | *ancora* | Kenting, Taiwan | BRCAS | AB441204-5 | AB441289-90 | - | - |
| Meandrinidae | *Meandrina* | *meandrites* | Bocas del Toro, Panama | UI | AB117295-6 [1] | AB117381 [1] | AB118431 [1] | - |
|  |  | *brasiliensis* | Abrolhos, Brazil | FURJ | AB11797 [1] | AB117382 [1] | AB118430 [1] | - |
|  | *Dendrogyra* | *cylindrus* | Portobelo, Panama | UI | AB117299 [1] | AB117384 [1] | AB118434-5 [1] | - |
|  | *Dichocoenia* | *stokesi* | Portobelo, Panama | UI | AB117298 [1] | AB117383 [1] | AB118433 [1] | - |
|  | *Eusmilia* | *fastigiata* | Bocas del Toro, Panama | UI | AB117294 [1] | AB117380 [1] | AB118432 [1] | - |
|  | *Ctenella* | *chagius* | Chagos Archipelago | no specimen | AB441208 | AB441293 | AB441378-9 | - |
| Siderastreidae | *Psammocora* | *contigua* | Yeliu, Taiwan | BRCAS | AB441209 | AB441294 | - | AY722783 |
|  | *Coscinaraea* | *columna* | Yeilu, Taiwan | BRCAS | AB441210 | AB441295 | AB441380-1 | - |
|  |  | *columna* | Aka Island, Okinawa, Japan | SMBL | - | - | - | AB441406 |
|  | *Siderastrea* | *siderea* | Bocas del Toro, Panama | UI | AB441211 | AB441296 | - | - |
|  |  | *radians* | Bocas del Toro, Panama | UI | AB441212 | AB441297 | AB441382-3 | - |
|  |  | *stellata* | Bocas del Toro, Panama | BRCAS | AB441213 | AB441298 | - | AB441407 |
|  |  | *savignyana* | Wanlitung, Taiwan | BRCAS | AB441214 | AB441299 | - | - |
|  |  | *savignyana* | Oman | BRCAS | AB441215 | AB441300 | - | - |
| Agariciidae | *Pavona* | *cactus* | Aka Island, Okinawa, Japan | SMBL | AB441216-7 | AB441301-2 | AB441384-5 | - |
|  |  | *cactus* | Taiwan | BRCAS | - | - | - | AB441408 |
|  | *Gardineroseris* | *planulata* | Aka Island, Okinawa, Japan | SMBL | AB441218 | AB441303 | - | - |
|  |  | *planulata* | Taiwan | BRCAS | - | - | - | AB441409 |
|  | *Agaricia* | *humilis* | Bocas del Toro, Panama | UI | AB441219 | AB441304 | AB441386 | - |
|  | *Leptoseris* | *cucullata* | Bocas del Toro, Panama | UI | AB441220-1 | AB441305-6 | AB441387-8 | - |
|  |  | *yabei* | Yeiliu, Taiwan | BRCAS | - | - | - | AB441410 |
|  | *Pachyseris* | *speciosa* | Aka Island, Okinawa, Japan | SMBL | AB441222 | AB441307 | AB441389 | - |
| Fungiidae | *Herpolitha* | *limax* | Aka Island, Okinawa, Japan | SMBL | AB441223 | AB441308 | AB441390 | - |
|  | *Fungia* | *scutaria* | Aka Island, Okinawa, Japan | SMBL | AB441224 | AB441309 | - | - |
|  | *Sandalolitha* | *robusta* | Aka Island, Okinawa, Japan | SMBL | - | - | - | AB441411 |
| Astrocoeniidae | *Stylocoeniella* | *guentheri* | Aka Island, Okinawa, Japan | SMBL | AB441225 | AB441310 | - | - |
|  |  | *guentheri* | Kenting, Taiwan | BRCAS | - | - | - | AY722793 |
|  | *Madracis* | *mirabilis#* | Bocas del Toro, Panama | UI | AB441226-7 | AB441311-2 | AB441391 | AB441412 |
|  | *Stephanocoenia* | *michelinii* | Bocas del Toro, Panama | UI | AB441228-9 | AB441313-4 | - | AB441413 |
| Pocilloporidae | *Pocillopora* | *verrucosa* | Aka Island, Okinawa, Japan | SMBL | AB441230 | AB441315 | AB441392 | - |
|  |  | *damicornis* | Kenting, Taiwan | BRCAS | - | - | - | AY722785 |
|  | *Stylophora* | *pistillata* | Penghu Island, Taiwan | BRCAS | AB441231 | AB441316 | - | AY722795 |
|  | *Seriatopora* | unidentified | Aka Island, Okinawa, Japan | SMBL | AB441232-3 | AB441317-8 | - | - |
|  |  | *hystrix* | Kenting, Taiwan | BRCAS | AB441234 | AB441319 | - | AY722794 |
| Dendrophylliidae | *Tubastraea* | *coccinea* | Kenting, Taiwan | BRCAS | AB441235-7 | AB441320-2 | - | AY722796 |
|  |  | unidentified | Aka Island, Okinawa, Japan | SMBL | AB441238 | AB441323 | - | - |
|  | *Dendrophylllia* | unidentified | Aka Island, Okinawa, Japan | SMBL | AB441239 | AB441324 | - | - |
|  | *Turbinaria* | *peltata* | Shirahama, Wakayama, Japan | SMBL | AB441240 | AB441325 | - | - |
| Poritidae | *Goniopora* | unidentified | Aka Island, Okinawa, Japan | SMBL | AB441241 | AB441326 | - | - |
|  |  | *columna* | Kenting, Taiwan | BRCAS | - | - | - | AB441414 |
|  | *Porites* | *astreoides* | Bocas del Toro, Panama | UI | AB441242 | AB441327 | - | - |
|  |  | *lutea* | Aka Island, Okinawa, Japan | SMBL | AB441243-4 | AB441328-9 | - | - |
|  |  | *lutea* | Penghu Island, Taiwan | BRCAS | - | - | - | AY722788 |
|  | *Alveopora* | unidentified | Kenting, Taiwan | BRCAS | AB441245 | AB441330 | - | - |
| Acroporidae | *Acropora* | *tenuis* | Magnetic Island, Australia | - | AF338425 [4] | AF338425 [4] | - | - |
|  |  | *palmata* | Bocas del Toro, Panama | BRCAS | AB441246 | AB441331 | - | - |
|  | *Isopora* [5] | *brueggemanni* | Togian Island, Indonesia | MTQ | AB441247 | AB441332 | - | - |
|  |  | *palifera* | Togian Island, Indonesia | MTQ | AB441248 | AB441333 | - | - |
|  |  | *togianensis* | Togian Island, Indonesia | MTQ | AB441249 | AB441334 | - | - |
|  | *Anacropora* | *matthai* | Togian Island, Indonesia | MTQ | AB441250 | AB441335 | - | - |
|  |  | *forbesi* | Togian Island, Indonesia | MTQ | AB441251 | AB441336 | - | - |
|  | *Montipora* | *cactus* | Penghu Island, Taiwan | MTQ | AB441252 | AB441337 | - | - |
|  | *Astreopora* | *myriophthalma* | Kenting, Taiwan | BRCAS | AB441253 | AB441338 | - | - |
|  |  | *explanata* | Kenting, Taiwan | BRCAS | AB441254 | AB441339 | - | - |
| Fungiacyathidae | *Fungiacyathus* | unidentified | Suao, Taiwan | BRCAS | AB441255 | AB441340 | - | AY722757 |
| Corallimorpharia | *Corynactis* | *californica* | Birch Aquarium at SIO | no specimen | AB441256-7 | AB441341-2 | - | AB441415 |
|  | *Pseudocorynactis* | unidentified | Birch Aquarium at SIO | no specimen | AB441258-9 | AB441343-4 | - | - |
|  | *Ricordia* | *florida* | Carrie Bow Cay, Belize | no specimen | AB441260 | AB441345 | - | - |
|  |  | *yuma* | Aka Island, Okinawa, Japan | no specimen | AB441261-2 | AB441346-7 | AB441393 | - |
|  | *Rhodactis* | *mussoides* | Kenting, Taiwan | BRCAS | AB441263 | AB441348 | - | - |
|  |  | *indosinensis* | Kenting, Taiwan | BRCAS | AB441264 | AB441349 | - | - |
|  |  | *bryoides* | Wanlitung, Taiwan | BRCAS | - | - | - | AB441416 |
|  |  | unidentified | Aka Island, Okinawa, Japan | no specimen | AB441265 | AB441350 | - | - |
|  | *Actinodiscus* | *nummiformis* | Yeilu, Taiwan | BRCAS | AB441266 | AB441351 | - | AB441417 |
|  | *Amplexidiscus* | *fenestrafer* | Kenting, Taiwan | BRCAS | AB441267 | AB441352 | - | AB441418 |
|  | *Discosoma* | *carlgreni* | Carrie Bow Cay, Belize | no specimen | AB441268 | AB441353 | - | - |
|  |  | unidentified | Aka Island, Okinawa, Japan | no specimen | AB441269-70 | AB441354-5 | AB441394 | - |
| Antipatharia | *Cirripathes* | unidentified | Kenting, Taiwan | BRCAS | AB441271-3 | AB441356-8 | AB441395 | - |
| Actiniaria | *Anemonia* | unidentified | Birch Aquarium at SIO | no specimen | AB441274 | AB441359 | - | - |
|  | *Stichodactyla* | unidentified | Carrie Bow Cay, Belize | no specimen | AB441275 | AB441360 | - | - |
|  | *Condylactis* | unidentified | Taiwan | BRCAS | - | - | - | AB441419 |
|  | *Metridium* | *senile* | - | - | AF000023 [6] | AF000023 [6] | - | - |
| Zoanthidea | *Zoanthus* | unidentified | Carrie Bow Cay, Belize | no specimen | AB441276-7 | AB441361-2 | - | - |
|  | *Sphenopus* | *marsupialis* | Suao, Taiwan | BRCAS | - | - | - | AB441420 |
| Octocorallia | *Heliopora* | *coerulea* | Taiwan | BRCAS | - | - | - | AB441421 |

**Goniastrea aspera* in [1]

**^†^***Oxypora lacera* in [1]

# Probably *Madracis* *auretenra* sensu Locke et al. [7]

BRCAS: Biodiversity Research Centre, Academia Sinica

SIO: Scripps Institution of Oceanography

SMBL: Seto Marine Biological Laboratory, Kyoto University

UI: University of Iowa

FMNH: Florida Museum of National History, University of Florida

FURJ: Federal University of Rio de Janeiro

MTQ: Museum of Tropical Queensland

1. Fukami H, Budd AF, Paulay G, Sole-Cava A, Chen CA, et al. (2004) Conventional taxonomy obscures deep divergence between Pacific and Atlantic corals. Nature 427: 832-835.
2. Lopez JV, Knowlton N (1997) Discrimination of the sibling species of the *Montastraea annularis* complex with multiple genetic loci. Proc 8^th^ Int Coral Reef Symp 2: 1613-1618.
3. Fukami H, Chen CA, Chiou C-Y, Knowlton N (2007) Novel group I introns encoding a putative homing endonuclease in the mitochondrial *cox1* gene of scleractinian corals. J Mol Evol 64: 591-600.
4. van Oppen MJH, Catmull J, McDonald BJ, Hislop NR, Hagerman PJ, et al. (2002) The mitochondrial genome of *Acropora tenuis* (Cnidaria; Scleractinia) contains a large group I intron and a candidate control region. J Mol Evol 55: 1-13.
5. Wallace CC, Chen CA, Fukami H, Muir PR (2007) Recognition of separate genera within *Acropora* based on new morphological, reproductive and genetic evidence from *Acropora togianensis*, and elevation of the subgenus *Isopora* Studer, 1878 to genus (Scleractinia: Astrocoeniidae; Acroporidae). Coral Reefs 26: 231-239.
6. Beagley CT, Okimoto R, Wolstenholme DR (1998) The mitochondrial genome of the sea anemone *Metridium senile* (Cnidaria): Introns, a paucity of tRNA genes, and a near-standard genetic code. Genetics 148: 1091-1108.
7. Locke JM, Weil E, Coates KA (2007) A newly documented species of [*Madracis*](http://www.itis.gov/servlet/SingleRpt/SingleRpt?search_topic=all&search_value=Madracis&search_kingdom=every&search_span=exactly_for&categories=All&source=html&search_credRating=All) (Scleractinia: Pocilloporidae) from the Caribbean. Proc Biol Soc Wash 120: 214-226.
