# Supplementary material for: Mitochondrial and Nuclear Genes Suggest that Stony Corals Are Monophyletic but Most Families of Stony Corals Are Not (Order Scleractinia, Class Anthozoa, Phylum Cnidaria)
Source: PLoS One. 2008 Sep 16;3(9):e3222. doi: 10.1371/journal.pone.0003222 (PMC2528942; doi:10.1371/journal.pone.0003222)
Supplement: Table S2 — Summary of possible changes to current taxonomy of reef-building corals [(1) for most corals, (2) for Fungiidae] and evidence supporting those changes. We list provisional placement based on mitochondrial data (from cox1 and cob from Fig. 1 unless otherwise noted); sources of additional evidence that supports the mitochondrial data are indicated in footnotes [some of these data also appeared in Fukami et al. (3)]. Note that not all members of speciose genera have been examined; in some cases these genera may ultimately be distributed among families, and we list species names where we know that different species have substantially different phylogenetic placements. This table suggests an outline for a revised taxonomy but does not represent a formal taxonomic revision. Families that are exclusively or almost exclusively azooxanthellate (Rhizangiidae, Caryophylliidae) are not included. In addition, the genera Blastomussa, Micromussa, Physogyra, and Plesiastrea are not included in lists of new affiliations because mitochondrial and nuclear data provide no consistent indication of likely close relatives. (0.10 MB DOC) [file pone.0003222.s002.doc]

SI Table 2. Summary of possible changes to current taxonomy of reef-building corals [(1) for most corals, (2) for Fungiidae] and evidence supporting those changes. We list provisional placement based on mitochondrial data (from *cox1* and *cob* from Fig. 1 unless otherwise noted); sources of additional evidence that supports the mitochondrial data are indicated in footnotes [some of these data also appeared in Fukami et al. (3)]. Note that not all members of speciose genera have been examined; in some cases these genera may ultimately be distributed among families, and we list species names where we know that different species have substantially different phylogenetic placements. This table suggests an outline for a revised taxonomy but does not represent a formal taxonomic revision. Families that are exclusively or almost exclusively azooxanthellate (Rhizangiidae, Caryophylliidae) are not included. In addition, the genera *Blastomussa*, *Micromussa*, *Physogyra*, and *Plesiastrea* are not included in lists of new affiliations because mitochondrial and nuclear data provide no consistent indication of likely close relatives.

| Traditional  families and new clades, with list of studied genera in the group based on mitochondrial data | Genera (at least in part) newly  embedded in or allied to family | Unstudied genera  (# species; H- only holotype available for genus) | Genera (at least in part) unrelated to type taxon or  majority of conventionally included genera | Likely future  status of family | Newly defined biogeographic distribution (taxa not distinctive at family level in parentheses) |
| --- | --- | --- | --- | --- | --- |
| **“Faviidae”**  *Cyphastrea*^a^  *Montastraea* (not  *cavernosa*)^a^  *Caulastraea*^a^  *Barabattoia*  *Favites*^a^  *Goniastrea*^a^  *Platygyra*^a^  *Oulophyllia*^a^  *Leptoria*^a^  *Echinopora*  *Favia* (Pacific)^a^ | *Trachyphyllia*^a^  *Pectinia*^a^  *Mycedium*^a^  *Merulina*^a^  *Scapophyllia*  *Hydnophora*^a^ | *Erythrastrea* (1)  *Australogyra* (1)  *Parasimplastrea* (2)  *Moseleya* (1) | *Leptastrea*^a, g, o^  *Oulastrea*^a, b, k^  *Solenastrea*^a, b, m^  *Cladocora* ^a, n^  *Plesiastrea*^a, t^  *Diploastrea*^j^  *Montastraea*  *cavernosa*^a^ | Type (*Favia fragum*) in newly defined Mussidae; new name required, perhaps several families | Mostly Pacific; only Atlantic taxa are members of the *Montastraea* *annularis* complex |
| **Mussidae**  *Scolymia* (Atlantic)^a^  *Mussa*^a^  *Mussismilia*^a^  *Isophyllia*^a^  *Mycetophyllia*^a^ | *Favia* (Atlantic)^a^  *Diploria*^a^  *Manicina*^a^  *Colpophyllia*^a^ | *Australomussa* (1)  *Indophyllia* (1H) | *Blastomussa*^a, u^  *Micromussa*^a, v^  *Acanthastrea*^a, i^  *Lobophyllia*^a, i^  *Symphyllia*^a, i^  *Cynarina*^a, i^  *Scolymia*  (Pacific)^a, i^ | Redefined to include only Atlantic members | Atlantic |
| **Merulinidae**  *Merulina*^a^  *Scapophyllia* | *Goniastrea*  *pectinata*^a^  *Leptoria*  *irregularis*^a^ | *Boninastrea* (1H) *Paraclavarina* (1) | *Hydnophora*^a^ | Not distinctive at family level, not monophyletic as originally delimited | (Pacific) |
| **Pectiniidae**  *Pectinia*^a^  *Mycedium*^a^ | *Caulastraea*^a^ *Oulophyllia*^a^ | *Echinomorpha* (1) | *Echinophyllia*^a^ *Oxypora*^a^ | Not distinctive at family level, not monophyletic as originally delimited | (Pacific) |
| **Trachyphylliidae**  *Trachyphyllia*^a^ | Some *Favia*^a^ | none (single genus) | none (single genus) | Not distinctive at family or subfamilial level | (Pacific) |
| **Meandrinidae**  *Meandrina*^a^  *Dichocoenia*^a^  *Dendrogyra*^a^  *Eusmilia*^a^ | none | *Gyrosmilia* (1)  *Montigyra* (1H) | *Ctenella*^a^ | Retained as family | Mostly or only Atlantic; two unstudied Pacific genera |
| **Oculinidae**  *Oculina*^a, b^ | *Solenastrea*^b, m^  *Cladocora*^a, n^ | *Simplastrea* (1)  *Schizoculina* (2) | *Galaxea*^a, r^ | Retained as family | Mostly or only Atlantic; one unstudied Pacific genus |
| **Fungiidae**  *Fungia*  *Sandalolitha*^b, c, d^  *Herpolitha*^a, d^  *Heliofungia*^c, d^  *Ctenactis*^c, d^  *Cycloseris* (incl.  *Diaseris*)^d^  *Polyphyllia*^d^  *Halomitra*^d^  *Zoopilus*^d^  *Podabacia*^d^  *Danafungia*^d^  *Lithophyllon*^d^  *Verrillofungia*^d^  *Lobactis*^d^  *Pleuractis*^d^ | *Leptastrea*^a, g, o^  (embedded)  *Psammocora*^b, g, p^  (allied)  *Coscinaraea*^b, g, p^  (allied)  *Oulastrea*^b, k^  (distantly allied)  *Horastrea*^e^  (allied)  *Anomastrea*^e^  (allied) | *Cantharellus* (3) | none | Retained as family | Pacific |
| **Pocilloporidae**  *Pocillopora*^b^  *Seriatopora*^b^  *Stylophora*^b^ | *Madracis*^b, g, q^  (allied)  *Stylocoeniella*^b^  (allied) | none | none | Retained as family | Pacific and Atlantic |
| **Dendrophylliidae**  *Turbinaria*  *Tubastraea* | none | *Duncanopsammia* (1)  *Rhizopsammia* (1)  *Balanophyllia* (2)  *Heteropsammia* (2)  Many azooxanthellate corals | none | Retained as family | Pacific and Atlantic |
| **Poritidae**  *Porites*^b^  *Goniopora*^b^ | none | *Stylaraea* (1)  *Poritipora* (1) | *Alveopora*^f, g, s^ | Retained as family | Pacific and Atlantic |
| **Fungiacyathidae**  *Fungiacyathus*^b^ | none | none (single genus) | none (single genus) | Retained as family | Pacific |
| **Siderastreidae**  *Siderastrea*^a^, ^b^  *Pseudosiderastrea*^e, not g^ | none |  | *Psammocora*^b, g, p^  *Coscinaraea*^a, b, g, p^ | Retained as family | Atlantic and Pacific |
| **Astrocoeniidae**  *Stephanocoenia*^b^ | none | *Paulastrea* (1) | *Madracis*^b, g, q^  *Stylocoeniella*^b^ | Retained as family (name pending status of fossil type) | Atlantic, perhaps Pacific; one unstudied Pacific genus |
| **Euphylliidae**  *Euphyllia*^a^ | *Ctenella*^a^  *Galaxea*^a, r^  *Pachyseris*^a, l^ (at least one spp distantly allied) | *Catalaphyllia* (1)  *Nemenzophyllia* (1)  *Plerogyra* (5) | *Physogyra*^a^ | Retained as family | Pacific |
| **Acroporidae**  *Acropora* ^f^  *Isopora*^f, h^  *Anacropora*  *Montipora*  *Astreopora*^f^ | *Alveopora*^f^, ^g, s^ | none | none | Retained as family | Atlantic and Pacific |
| **Agariciidae**  *Agaricia*^a^  *Pavona*^a, b^  *Gardinoseris*^b^  *Leptoseris*^a, b^ | none | *Coeloseris* (1) | *Pachyseris*^a, l^ | Retained as family | Atlantic and Pacific |
| **New clade**  *Acanthastrea* ^a, i^  *Echinophyllia*^a^  *Oxypora*^a^  *Lobophyllia* ^a, i^  *Symphyllia* ^a, i^  *Cynarina* ^a, i^  *Scolymia*  (Pacific) ^a, i^ | NA | NA | NA | New family or clade of related families | Pacific |
| **New clade**  *Diploastrea*^b, j^ | NA | NA | NA | Candidate for single-genus family status | Pacific |
| **New clade**  *Montastraea*  *cavernosa*^a^ | NA | NA | NA | Candidate for single-genus family status | Atlantic |
| **New clade**  *Oulastrea*^a, b, k^ | NA | NA | NA | Possible candidate for single-genus family status or subfamily within Fungiidae | Pacific |
| **New clade**  *Pachyseris*^a, l^ (not all spp.) | NA | NA | NA | Possible candidate for single-genus family status or subfamily within the Euphylliidae | Pacific |

^a^ Fig. 2 (tubulin), this paper

^b^ Fig. 3 (ribosomal), this paper

^c^ COI analyses demonstrate close relationships to other fungiids in Fig. 1 (data not shown).

^d^Gittenberger et al. (2)

^e^Benzoni et al. (4)

^f^ Wei et al. (5)

^g^ Kerr (6, which summarizes many morphological and molecular studies, including especially Romano and Cairns 2000, Le Goff-Vitry 2004)

^h^ *Isopora* is distinguished from *Acropora* by its supplementary axial corallite, brooding of planula larvae and genetic distinctness. Wallace et al. (7) proposed the elevation of the subgenus *Isopora* to genus.

^i^ *Acanthastrea*, *Lobophyllia*, *Symphyllia*, *Cynarina*, and Pacific *Scolymia* have septal teeth and ornamentation that differ in shape from Atlantic Mussidae (8). They are constructed by weak secondary calcification axes, which are surrounded by extensive thickening deposits. Wells (9) placed the Pacific *Scolymia* in different genera (*Homophyllia*, *Parascolymia*) from Atlantic *Scolymia*, because of lamellar linkage among corallite centers during budding and/or numerous thick granulations on septal faces.

^j^ *Diploastrea* has the following morphologic features that are inconsistent with the “Faviidae”: synapticulae; septal teeth composed of compound trabeculae and extensive thickening deposits (forming concentric rings).

^k^ *Oulastrea* has the following morphologic features that are inconsistent with the “Faviidae”: synapticulae, papillose columella, paliform lobes. It was placed in the Agariciidae by Vaughan and Wells (10).

^l^ *Pachyseris* has the following morphological features that are inconsistent with the Agariciidae: indistinct centers, laminar columella.

^m^ *Solenastrea* has the following morphological characters that are inconsistent with many faviids and consistent with the Oculinidae: non-costate, vesicular coenosteum; well-developed paliform lobes.

^n^ *Cladocora* has the following morphological characters that are inconsistent with many faviids and consistent with the Oculinidae: papillose columella, well-developed paliform lobes.

^o^ *Leptastrea* has the following morphological features consistent with the Fungiidae but inconsistent with the “Faviidae”: papillose columella, minute septal teeth composed of compound trabeculae, dense coenosteum, absent endotheca. However, *Leptastrea* lacks synapticulae, which are characteristic of the Fungiidae. Alloiteau (11) described the genus as transitional between the families “Faviidae”, Oculinidae, and Rhizangiidae.

^p^ *Psammocora* and *Coscinaraea* were placed in the Siderastreidae (as defined by *Siderastrea*) because of their synapticulae and septal fusion around the columella fossa. They have the following morphological features consistent with the Fungiidae but inconsistent with the Siderastreidae: intramural budding, multidirectional septal teeth formed by compound trabeculae (versus simple trabeculae in *Siderastrea*), and enclosed entosepta. Some species also have fulturae (=compound synapticulae) (4). However, *Psammocora* and *Coscinaraea* lack fenestrate septa, which are characteristic of the Fungiidae.

^q^*Madracis* was questionably placed in Pocilloporidae by Vaughan and Wells (10) and Wells (12) based on its well-developed coenosteum.

^r^ *Galaxea* is distinguished from *Oculina* by its vesicular coenosteum and weak columella. Vaughan and Wells (10) placed *Galaxea* and *Oculina* in separate subfamilies within the Oculinidae, based on the structure of the coenosteum and colony form. *Galaxea* is similar to *Euphyllia* in its weak/absent columella and the shape of its septal teeth.

^s^ *Alveopora* has the following morphological features consistent with the Acroporidae but inconsistent with the Poritidae: septa composed of simple trabecular spines projecting horizontally from mural “trabeculae”, columella weak or absent. However, *Alveopora* lacks extensive coenosteum, which is characteristic of the Acroporidae.

^t^ As characterized by Alloiteau (11), *Plesiastrea* has the following morphologic features that are inconsistent with the “Faviidae”: well-developed pali, papillose columella.

^u^ *Blastomussa* has the following morphological features that are inconsistent with Pacific “Mussidae”: extramural budding, polyps lacking organic connection in the adult stage, and papillose columella. The type species *Blastomussa merletti* (Wells, 1961) was originally placed in the Oculinidae (Subfamily Galaxeinae).

^v^ *Micromussa* was erected by Veron (1) for members of the genus *Acanthastrea* that have small corallites. It has the following morphological features that are inconsistent with Pacific “Mussidae”: paliform lobes.

References

1. Veron JEN (2000) *Corals of the World*. Australian Institute of Marine Science, Townsville.

2. Gittenberger A, Reijnen BT, Hoeksema BW (2006) in Gittenberger A, *The Evolutionary History of Parasitic Gastropods and their Coral Hosts*. PhD Dissertation, Leiden Univ., pp 37-56.

3. Fukami H, Budd AF, Paulay G, Sole-Cava A, Chen CA, Iwao K, Knowlton N (2004) *Nature* 427:832-835.

4. Benzoni F, Stefani, F, Stolarski J, Pichon M, Mitta G, Galli P (2007) *Contr Zool* 76: 35-54.

5. Wei WV, Wallace CC, Dai CF, Moothien Pillay RK, Chen CA (2006) *Zool Stud* 45:404-418.

6. Kerr AM (2005) *Biol Rev* 80: 543-558.

7. Wallace CC, Chen CA, Fukami H, Muir PR (2007) *Coral Reefs* 26:231-239.

8. Budd AF (2006) *Geol Soc Am Abs Prog* 38 (7): 534.

9. Wells JW (1964) *Zoöl Meded*, Leiden, 39: 375-384.

10. Vaughan TW, Wells JW (1943) *Geol Soc Am Spec Pap* 44:1-363, pl.1-51.

11. Alloiteau J (1957) *Contribution a la systématique des madréporaires fossiles*. CNRS, Paris, 1-462, 286 figs, 20 pls.

12. Wells JW (1956) in Moore, RC (ed.), *Treatise on Invertebrate Paleontology*. Geol Soc Am and U Kansas Press, Lawrence, KS, pp. F328-444.
